# Effects of Temperature on the p53-DNA Binding Interactions and Their Dynamical Behavior: Comparing the Wild Type to the R248Q Mutant

**DOI:** 10.1371/journal.pone.0027651

**Published:** 2011-11-16

**Authors:** Khaled Barakat, Bilkiss B. Issack, Maria Stepanova, Jack Tuszynski

**Affiliations:** 1 Department of Physics, University of Alberta, Edmonton, Alberta, Canada; 2 Department of Engineering Mathematics and Physics, Fayoum University, Fayoum, Egypt; 3 Department of Electrical and Computer Engineering, University of Alberta, Edmonton, Alberta, Canada; 4 National Institute for Nanotechnology, National Research Council, Edmonton, Alberta, Canada; 5 Department of Oncology, University of Alberta, Edmonton, Alberta, Canada; Semmelweis University, Hungary

## Abstract

**Background:**

The protein p53 plays an active role in the regulation of cell cycle. In about half of human cancers, the protein is inactivated by mutations located primarily in its DNA-binding domain. Interestingly, a number of these mutations possess temperature-induced DNA-binding characteristics. A striking example is the mutation of Arg248 into glutamine or tryptophan. These mutants are defective for binding to DNA at 310 K although they have been shown to bind specifically to several p53 response elements at sub-physiological temperatures (298–306 K).

**Methodology/Principal Findings:**

This important experimental finding motivated us to examine the effects of temperature on the structure and configuration of R248Q mutant and compare it to the wild type protein. Our aim is to determine how and where structural changes of mutant variants take place due to temperature changes. To answer these questions, we compared the mutant to the wild-type proteins from two different aspects. First, we investigated the systems at the atomistic level through their DNA-binding affinity, hydrogen bond networks and spatial distribution of water molecules. Next, we assessed changes in their long-lived conformational motions at the coarse-grained level through the collective dynamics of their side-chain and backbone atoms separately.

**Conclusions:**

The experimentally observed effect of temperature on the DNA-binding properties of p53 is reproduced. Analysis of atomistic and coarse-grained data reveal that changes in binding are determined by a few key residues and provide a rationale for the mutant-loss of binding at physiological temperatures. The findings can potentially enable a rescue strategy for the mutant structure.

## Introduction

P53 is the most mutated protein in human cancers,[1] and mutations of p53 alone account for more than half of invasive types of cancer.[2] According to the latest version (R15) of the *TP53* mutation database,[3] 27 580 different somatic mutations have been identified in the full-length protein and the overwhelming majority of alterations are located within the core DNA-binding domain (DBD). More importantly, ∼75% of the resulting mutants are, fundamentally, full-length proteins with single amino acid substitutions in the DBD. In addition, about 40% of the DBD mutations are concentrated at six particular hot-spots: Arg-175, Gly-245, Arg-248, Arg-249, Arg-273 and Arg-282.[4]

Of the six hot-spot residues listed above, alterations at Arg-248 and Arg-273 are classified as DNA contact mutations whereas substitutions at the remaining sites are structural mutations. Contact mutants are characterized by the direct loss of the sequence–specific transactivation activity while retaining the wild-type (WT) conformation.[5] Structural mutations, on the other hand, involve residues primarily responsible for maintaining the conformational integrity of the DBD and stabilizing the p53 DNA–binding surface. Such alterations generate local structural defects, which in turn transfer to critical regions of the DBD, causing indirect loss of DNA binding.[6] Failure to bind DNA prevents p53-dependent transcription and hence inhibits p53-mediated tumor suppression.

Investigations of the thermodynamic stability of the DBD have revealed the destabilizing nature of hot-spot mutations relative to WT p53[7],[8],[9] and highlighted the temperature-dependence of their DNA-binding affinity.[8],[10] Other studies focused on different thermodynamical parameters that can determine the ultimate stability of the protein and its mutants. Such experiments included measuring pressure-stability at different temperatures [11],[12],[13], different pHs [14] and studying the effect of DNA-binding on the core domain stability.[15]

The first insightful evidence for the importance of temperature in proper DNA binding was reported by Zhang and collaborators in 1994 for Ala-143, which was considered a hot-spot mutation at the time.[10] The mutant p53 exhibited high DNA-binding affinity at temperatures of 306 K and below, as well as stronger transcriptional activity than WT p53. At physiological temperature both the DNA-binding and transcriptional activation functions of the mutant were significantly reduced. These observations were rationalized in terms of a two-conformational state model: a mutant conformation at physiological temperatures, and a wild-type conformation at lower temperatures. Friedlander *et al.* examined the effects of temperature on a wide range of p53 mutants.[16] This included Ala-143, His-175, Trp-248, Ser-249 and His-273. With the exception of His-175, all mutants were able to bind DNA at sub-physiological temperatures (298–306 K). At 310 K, however, their binding to DNA was defective. Numerous other temperature-sensitive mutations were later identified and targeted for restoration.[17] Ishioka's group alone assessed a collection of over 2,000 p53 mutants for temperature sensitivity and identified 113 mutants with activity at 303 K.[18] This represents about ∼10% of all reported single amino acid alterations of the DBD in human cancers.[3] Here, we focused on the R248Q mutant, which is the most frequently occurring mutation in human cancer. It is mostly associated with breast, colon, head, neck and skin cancers. Moreover, it ranks as the second most mutants in esophageal, gastric, lung, ovarian, and prostate cancers.

In this paper, we report on the results of molecular dynamics (MD) simulations that have been carried out for the DBD of WT and R248Q p53 molecules in the presence or absence of a DNA duplex at 300, 305 and 310 K. A comprehensive assessment of the influence of temperature on p53-DNA intermolecular interactions has been performed in terms of structural, dynamical and thermodynamic properties. Because macromolecular binding is often accompanied by large structural rearrangements, the slow large-amplitude motions of p53 molecules and their DNA complexes have also been analyzed using the essential collective dynamics method.[19] The main aims of this work are to determine the effects of temperature on the conformations of WT and mutant p53 complexes and to identify key residues or regions of the complexes, which modulate changes in DNA-binding at the different temperatures. Our results indicate that temperature plays an essential role in the stability of the hydrogen bond network and binding properties of p53-DNA complexes over both short and long time-scales. The outcome of our study provides new insights into the way towards restoring apoptosis in the above-mentioned types of cancer cells by activating the p53 pathway of tumorigenic R248Q mutants.

## Results and Discussion

Mutations at the Arg-248 residue of p53 have been of substantial interest to a large group of cancer researchers. Many experiments were conducted in order to better understand their roles. With the objective of restoring the activity of mutated p53 proteins, many researchers employed various experimental and theoretical techniques aimed at understanding why they are inactive in cancer cells.

The work presented here was inspired by many experimental studies that focused on the effects of temperature on the stability, structure and transcriptional activity of p53 and its Arg-248 mutants. For example, Bullock *et al.* investigated the wild-type stability along with a number of its mutants including R248Q at both low and high temperatures [7]. Their work revealed that the R248Q mutant is stable at sub-physiological low temperatures. The R248Q stability was less than that of the wild-type protein by ∼2 kcal/mol. The mutant structure also retained a two-stage unfolding transition, similar to the wild-type protein [11], which indicated well-defined structures at low temperature. Interestingly, the addition of a 22-mer double-stranded DNA p53 consensus sequence raised the melting temperature of the tested proteins, signifying a stabilizing effect due to DNA-binding [7]. The effect of DNA on stabilizing the core domain was recently confirmed by Ishimaru *et al.* [15]. An interesting study by Wong *et al.* investigated the structural changes introduced by five hot spot mutations including R248Q at low temperature using chemical shift changes [20]. Their findings indicate that the R248Q mutation induces structural changes in L2 and L3 regions of the core domain at 310 K. That is, the R248Q mutation has the dual capacity of being both a contact and a structural mutation. These structural changes lower the binding affinity to the DNA without significantly destabilizing the protein [21]. In fact, at high pressure and low temperature, WT p53 can adopt the R248Q mutant structure [11]. Benoit *et al.* investigated the transcriptional activation of cyclooxygenase-2 (Cox-2) by p53 at low temperature [22]. They also examined Cox-2 transcription induced by different p53 mutants including the R248Q variant. Cooperating with nuclear factor-kappaB (NF-kappaB), R248Q produced a significant increase in Cox-2 expression similar to the wild-type protein.

Other common mutations of the Arg-248 residue (e.g. R248W and R248A) also expressed a profound dependence on temperature. The most perceptible behavior was noticed in the case of R248W [16], [18]. Friedlander *et al.* [22] showed that R248W can effectively bind to DNA at low temperatures and this binding activity is significantly diminished at physiological temperatures. A kinetic stability experiment on a number of different p53 mutants revealed that R248A had a half-life time (*t*_1/2_) of 128 minutes at low temperature compared to less than 3 minutes at 310 K. The analysis in this study revealed an important concept in understanding the stability of p53 mutants. Namely, there is a remarkable correlation between the thermodynamic and kinetic instability of the mutants. The more unstable the mutant, the shorter its half-life time. This means that R248A is more stable at low temperatures than at physiological temperatures. All of the above-mentioned experimental data reveal a clear connection between temperature and the stability and activity of p53 R248 mutants in general and the R248Q mutant in particular.

### MD Simulations Of The Wild Type And Mutant Structures

**Figure 1:**
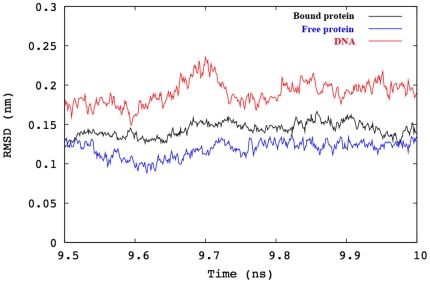
Plots of backbone RMSD for the DNA-bound and DNA-free WT p53 at 300 K over the final ns of 10 ns trajectories. The black, blue and red lines correspond to RMSD values of the DBD bound to DNA, in absence of DNA, and of DNA only, respectively.

The root mean square deviations (RMSD) of backbone atoms of the DBD and DNA duplex (for the p53-DNA complexes) were computed over the final ns of each trajectory. The results are shown in figure 1 for the wild type at 300 K. In the rest of the cases the behavior was similar (data not shown). The RMSDs of DNA are significantly higher and are associated with larger fluctuations than those of the protein in all trajectories. The higher mobility of the DNA backbone relative to the protein backbone in both complexes at all temperatures can be attributed to the dynamics of the terminal residues of the double helix that are not bound to the DBD. The RMSD plots of DNA-bound and DNA-free proteins are generally similar. The mean RMSD of the DBD is slightly smaller in the p53-DNA complexes than in the apo-structures for both p53 variants. Similar observations were reported by Noskov *et al.* [23] for the same protein at 300 K. In general, the backbone RMSDs appear to be relatively stable to temperature changes over the range investigated.

### Hydrogen Bonding and Water Distribution

Several reports of the crystal structure of the p53-DNA complexes have highlighted the central roles of water molecules and hydrogen bonds in stabilizing interactions between the two biomolecules. In some of these investigations, the failure of p53 to bind DNA has been correlated with the loss of one or two hydrogen bonds mediated by a single residue within the DBD. For example, the mutations of the hot-spot residue Arg-273[24] or Arg-249[2] into histidine or serine respectively, induces a sequence of hydrogen bond disruptions that ultimately lead to the loss of DNA binding. For these two mutations, the hydrogen bond network could be restored or compensated by means of an additional single mutation. Changing the 284-residue to arginine conferred DNA-binding ability to the R273H mutant. Similarly, substitution of the residue at position 268 by arginine partially restored the activity of the R249S mutant. The influence of hydration on p53 folding has been studied by Silva *et al.* and revealed that water interactions with both p53 and the DNA were essential for proper folding and enhanced stability of the complex [13]. The presence of a DNA molecule augmented the stability of the DBD within p53 [15]. Below we confirm these concepts by investigating the dynamical character of the hydrogen bond network. We also compare the different connections among the protein, DNA and water residues for both the wild type and the R248Q mutant at all temperature ranges investigated.

**Figure 2:**
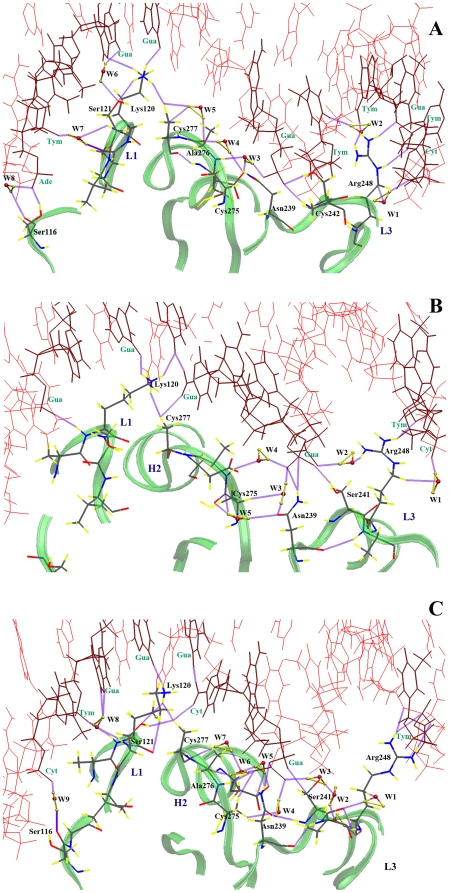
Hydrogen bond network for the wild type at three different temperatures. (A) 300 K, (B) 305 K and (C) 310 K.

**Figure 3:**
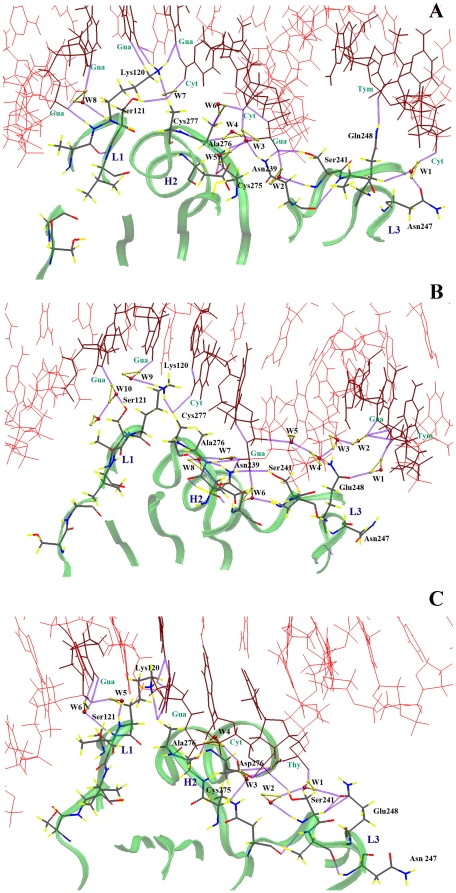
Hydrogen bond network for the Q248 mutant at three different temperatures. (A) 300 K, (B) 305 K and (C) 310 K.

Figures 2 and 3 describe the complicated hydrogen bond networks formed by interfacial atoms in the dominant structures extracted from clustering of the MD simulations. In figure 2-A, several direct contacts can be identified between the DBD of WT p53 and DNA nucleotides at 300 K. Arg-248 from the loop L3 protrudes into the minor groove of the DNA molecule resulting in favorable electrostatic interactions between the positively charged guanidinium group of Arg-248 and the negatively charged DNA backbone. The minor groove adjacent to Arg-248 is compressed and its bases are buckled so that the side chain of Arg-248 makes three direct contacts with the DNA. Likewise, the side-chains of Cys-242, Lys-120 and Ser-116 directly interact with DNA. Within the protein structure, Cys-277 is hydrogen-bonded to the side chain of Lys-120. In addition to direct p53-DNA contacts, seven ordered water molecules are located at the interface. Among these water molecules are conserved crystallographic water molecules present in the original crystal structure, thus supporting their inclusion in the starting structures for MD simulations. For clarity, only water molecules participating in the hydrogen bond network and which act as linkers between the different interacting residues are depicted in the figures. Water molecules appear to have a stabilizing role on the direct p53-DNA contacts. W1 and W2 connect Arg-248 to DNA through three different hydrogen bonds. W3 mediates an interaction between the side chain of Asp-281 and the backbone of Ala-276 while at the same time connecting them to the guanine base of DG-303. W4 and W5 are involved in water-bridged hydrogen bonds linking Asn-239 to Cys-277 and Ala-276 to Cys-277, respectively. W6 and W7, on the other hand, are responsible for maintaining a hydrogen bond network through which Ser-121 interacts with the DNA molecule via two different hydrogen bonds. Among the residues identified in the vicinity of DNA, Lys-120 and Ser-121 have been suggested as key participants in DNA binding in a crystallographic analysis of DNA-bound and DNA-free forms of the WT DBD.[25] In addition, p53 DBD lacking residues 100–120 displayed reduced binding during antibody binding experiments.[26]

Raising the temperature to 305 K does not significantly alter the overall structure of the protein-DNA binding interface or its hydrogen bond network (see figure 2-B). Arg-248 has retained one of the direct contacts and two water-bridged hydrogen bonds (W1, W2) with the DNA molecule. A direct contact between DNA and Ser-241, that was absent at 300 K, is present at 305 K alongside nucleotide-interactions with Asn-239, Cys-277 and Lys-120. Moreover, the water molecules W3, W4 and W5 mediate interactions between Asn-239, Cys-275 and DNA. At 310 K, interactions between Arg-248 and DNA amount to four hydrogen bonds and no water-bridged linkages are present, as shown in figure 2-C. However, W1 is involved in connecting Arg-248 and Ser-241, which in turn interacts with DNA nucleotides through W2 and W3. Cys-275 is connected to DNA through its side chain and through W4. Cys-277 is involved in an extensive hydrogen bond network via backbone interactions with the side chain of Asn-239 mediated by W5, W6 and W7 and side-chain interactions with DNA and Lys-120. Moreover, Ser-121 and Ser-116 are connected to DNA through the two water molecules; W8 and W9. Finally, similar to the previous cases, Lys-120 maintains its direct connection to DNA and to Cys-277. These strong interactions and persistence of the native fold of p53 DBD confirmed the fact that the wild type is stable at all three temperatures [7]. Interactions with the DNA molecule are extremely favored by the protein. They enhance its stability and prevent it from misfolding or aggregating [15]. Armed with the validation of our protocol in reproducing the native p53 conformation and constructing a fine grid of detailed hydrogen bonding interactions, we proceeded to investigate in detail the R248Q case.

Switching to the mutated p53 structure has yielded interesting findings. figure 3-A illustrates the hydrogen bond network at 300 K. Glu-248 is connected to DNA through a direct hydrogen bond and a water-mediated hydrogen bond. This water molecule, W1, also connects Glu-248 to Asn-247, which was absent in WT p53 at any of the three temperatures. Once more, water molecules play a major role in coordinating a number of hydrogen bonds at the DNA-p53 binding interface. For example, W2 connects Ser-241 to the DNA molecule. W3 and W5 connect Asn-239 to Cys-275. W4 and W6 connect the backbone of Ala-276 to the guanine residue DG-303 and its side chain to the cytosine residue DC-304, respectively. W8 mediates a superior interaction between the backbone of Lys-120 and guanine residue DG-318. In addition, the side chain of Ser-241 is hydrogen bonded to the side chain of Asn-239, and as observed for WT p53, Cys-277 preserves its hydrogen bond with Lys-120, which maintains direct contact with DNA. At 305 K (see figure 3-B) an important modification takes place. Glu-248 loses its direct contact with the DNA molecule as the distance between the side chain of Glu-248 and the closest DNA residue is greater than 4 Å. This results in a large gap between the protein and DNA and leads to a distortion in the minor groove close to Glu-248. Despite this deviation, Glu-248 participates in two hydrogen bonds with DNA through two water molecules, W1 and W4. A fine hydrogen bond network connects guanine DG-324 through four water molecules (W2, W3, W4 and W5) to guanine DG-303 in the middle of the DNA. This final guanine residue is directly connected to Ser-241, Asn-239 and Ala-276. Surprisingly, Cys-276 maintains its interaction with Lys-120, which was connected to DNA through a water molecule, W9, unlike the previously mentioned cases where Lys-120 had a direct contact to the DNA. Finally, Ser-121 is connected to DNA through W10. A huge difference is found to occur at 310 K (see figure 3-C). The separation between Glu-248 and DNA is more than 6 Å. No direct hydrogen bonds are established to connect Glu-248 to DNA. The DNA terminal near Glu-248 is completely distorted and separated from the protein. However, Glu-248 is connected to the center of the DNA duplex through a water-mediated hydrogen bond (W1) that also links Ser-241 to DNA. Cys-275 is connected to DNA through W3. Ala-276 is hydrogen bonded to Lys-120 while Ser-241 is attached to DNA via two water-mediated hydrogen bonds.

The aforementioned atomistic details of the DNA-contact geometry reveal a reasonable dependence on temperature. The L3 loop was directly linked to the minor groove of the bound DNA via Arg-248 at three different temperatures for the wild type, or via Glu-248 at 300 K for the R248Q mutant. During the six different simulations, the conformations of R/Q248 side chain were fully extended and contacted the DNA nucleotides either directly or indirectly through water molecules. It has also become apparent that the L3 loop plays a dual role in DNA binding. Besides contacting DNA through Arg-248, it is also an essential part of the DBD of p53 by aiding in the stabilization of the zinc-binding site and hence can affect other regions of the protein. Although the minor groove area is largely affected upon the mutation at physiological temperatures, the major groove contacts, *i.e.*, Lys-120, Ala-276 and Cys-277 maintain their interactions with DNA even after Arg-248 was mutated to glutamine.

In addition to the well-documented stabilizing roles of water-mediated interactions in biological complexes, hydration can also have a destabilizing effect, as recently described by Silva *et al.* [15] The authors attributed the enhanced stability of cognate DNA-WT p53 complexes to the exclusion of unfavorable water-mediated interactions from the protein surface. Conversely, infiltration of water inside mutant complexes would be responsible for their destabilization and promote aggregation of p53 molecules. The above reasoning suggests that the lower stability of the R248Q p53 complex at high temperatures is a result of structural changes in its hydration networks, as evidenced by the formation of an interfacial water-filled cavity at 305 K and the transformation of direct DNA contacts into water-mediated interactions at 305 and 310 K. Therefore, alterations in the hydrogen bond network provide an effective structural framework for understanding changes in DNA binding for the R248Q mutant p53 at physiological temperatures.

### Binding Energy Analysis

MD simulations of the p53–DNA complexes and the hydrogen bonding analysis provided valuable insights into the dynamics of their interactions and the role of water at the interface of complexes. Our next step was to investigate the influence of temperature on the stability of the p53 variants. To this end, the thermodynamics of p53-DNA binding were evaluated using the molecular mechanics Poisson-Boltzmann surface area (MM-PBSA) method, a well-established technique that takes into account the effects of solvation, ionic concentrations, entropy and molecular mechanics interactions. It has been previously employed in many similar studies[27],[28]_,_ [29] and has produced accurate free energy estimates at a reasonable computational cost. Its main advantages include the lack of adjustable parameters and the possibility of using a single MD simulation for the complete system to determine all energy values.

The binding energy calculations are listed in Table 1 for the two p53 structures at three different temperatures. It should be mentioned that binding energies are reported relative to the WT binding energy at 300 K, which was estimated as −12 kcal/mol. Our calculations indicate that binding to DNA is maintained by the WT protein both at 305 and 310 K. This is supported by experimental evidence that WT p53-complex has a melting temperature of 322 K, indicating that the complex is stable at 310 K.[7] While our results indicate that the binding affinity is enhanced at physiological temperature, *in vitro* measurements showed a decrease in the binding affinity of WT p53 at 310 K.[16],[30] The conflicting observations may be related to experimental conditions and techniques. It has been shown that the stability of p53 and DNA binding affinity is highly sensitive to ionic strength, DNA sequence and pressure.[31],[16] Nonetheless, the results agree on the qualitative aspects of binding, *i.e.*, WT p53 DBD can bind to the DNA at all three temperatures and also validates the MM-PBSA method as an adequate binding energy evaluation technique.[16] At 300 K, the binding energy of R248Q is decreased by ∼3 kcal/mol compared to the WT at the same temperature, signifying its possible binding to DNA. When the temperature is raised to 305 K and 310 K, the binding energy of the mutant p53 increases by 12 and 15 kcal/mol, respectively, relative to the WT. These observations indicate that binding of R248Q to DNA becomes highly unfavorable with increasing temperature. Taken together with our observations from hydrogen bond analysis, changes in the binding energy of the mutant p53 may be interpreted as a significant weakening of DNA-binding at 305 and 310 K while WT p53 retains its binding characteristics at these same temperatures.

**Table 1:** Binding energy changes between DNA and the p53 core domain due to temperature alterations.

Type	T (K)	BE_T_-BE_WT300K_ (kcal/mol)±1
WT	300	0
	305	3
	310	−12
R248Q	300	3
	305	12
	310	15

Note: BE_WT300K_ = −12 kcal/mol.

All binding energies are relative to that of the WT at 300 K. Our calculations predict that the WT p53 maintains its DNA binding at all temperatures. On the other hand, while the Glu-248 mutant (R248Q) does not lose its DNA binding activity at 300 K, binding is highly unfavorable at 310 K.

To further identify the regions of the protein that cause the loss of DNA binding, we decomposed the binding energy into residue contributions. Table 2 lists the individual contributions of residues that amount to at least ±1 kcal/mol of the binding energies, computed at 300, 305 and 310 K for the WT and R248Q p53. Again, the reported binding energies are relative to the WT at 300 K. Comparing the WT to the mutant p53, as expected, the substitution of arginine to glutamine carries the largest penalty which is associated with a cost of ∼8 kcal/mol at all temperatures. The residues Ser-241 and Asn-239, which are close to the mutation site, reduce the binding energy by ∼4 kcal/mol. This loss of binding energy is balanced by gains at the 119, 120, 276 and 277 sites.

**Table 2:** Binding energy decomposition per residue for WT and R248Q p53-DNA complexes at 300, 305 and 310 K.

Residue	BE_WT_-BE_WT300K_ (kcal/mol)			BE_RQ_-BE_WT300K_ (kcal/mol)		
	Temperature (K)			Temperature (K)		
	300	305	310	300	305	310
119	0	−3	−5	−5	−1	−4
120	0	1	−4	−3	2	−1
122	0	1	0	2	1	2
174	0	0	1	0	0	1
180	0	−1	−1	0	0	−1
184	0	0	−1	0	−1	−1
239	0	2	3	0	−1	2
240	0	0	0	0	0	1
241	0	0	0	1	1	2
243	0	0	0	−2	−1	0
248	0	1	−2	8	9	8
273	0	0	0	1	3	1
275	0	0	−1	−2	−1	0
276	0	−1	−2	−1	−2	−1
277	0	−2	−1	−2	−1	−2
ZN+2	0	0	0	−1	−1	0
DNA300	0	0	0	1	0	0
DNA301	0	−1	0	0	1	0
DNA302	0	2	2	1	3	3
DNA303	0	2	0	0	−1	2
DNA304	0	0	−3	−2	−2	−2
DNA315	0	−1	−1	−1	−1	−2
DNA306	0	−1	1	0	0	−1
DNA317	0	−1	−3	2	1	2
DNA318	0	2	3	3	2	3
DNA319	0	0	0	0	1	0
DNA320	0	−2	0	−2	−1	−2
DNA324	0	1	1	3	2	2
DNA325	0	2	1	2	1	1
DNA326	0	1	0	0	0	1

Binding energies are given relative to the energy of the DNA−bound WT p53 complex at 300 K. Residues 119, 120, 248 and 277 from p53 contributed the most to temperature-induced changes in binding energy. At least eight DNA residues involved in close contacts with the protein contributed significantly to binding.

Comparing these findings to the hydrogen bond analysis mentioned earlier reveals an outstanding correspondence. The stability of the hydrogen bond network at the three different temperatures in the wild-type protein indicates an unremitting binding to DNA. On the other hand, the lack of strong hydrogen bonding in the mutant variant at higher temperatures, namely 305 K and 310 K, causes a parallel effect on the binding affinity to DNA. In general, our analysis reveals that temperature-sensitive residues are located in the three loops and in the C-terminal region. The substitution of arginine by glutamine at residue 248 leads to changes in binding far from the mutation site, particularly in loop L1. This is consistent with differences observed in the major groove contacts during hydrogen bond analysis, and with the proposed classification of the R248Q as a dual structural/contact mutant.[14],[20] In addition, the zinc ion contributed significantly to the overall binding energy between the protein and DNA in all simulations. These energies ranged from −7 kcal/mol for the WT to −8 kcal/mol for the mutant p53 at 300 K. These results are consistent with the findings of Butler *et al.* that zinc is crucial for proper DNA binding, and that the stability of the zinc ion within the R248Q mutant is quantitatively comparable to that of the WT protein.[21]

### Essential Collective Dynamics

Macromolecular binding often leads to slow conformational rearrangements, which cannot be probed directly by conventional MD methods. To gain insight into the slow dynamics of DNA-binding of p53 molecules, we have explored the collective dynamical behavior of WT and R248Q p53 in the DNA-bound and free forms using a novel approach [19]. Based on the theory of collective dynamics, the method is a robust numerical technique with the ability to predict *slow* macromolecular motions from relatively short molecular dynamics trajectories. It has been successfully applied to characterize the global dynamics and flexibility of protein G[19] and prion proteins [32],[33] and has been employed in the present work to probe the dynamics of *long-lived* p53-DNA interactions in the present work.

**Figure 4:**
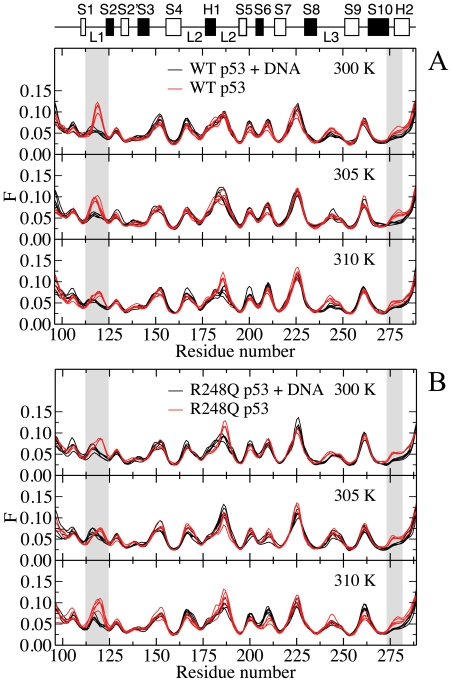
Main-chain flexibility profiles of (A) WT p53 and (B) R248Q p53 at various temperatures. Flexibility profiles determined from Cα atoms of DNA-bound (black solid line) and DNA-free (red solid line) WT and R248Q p53 at 300, 305 and 310 K. Regions of interest (loop L1 and loop between S10 and H2) are highlighted in grey.

The main-chain flexibility profiles of DNA-bound and DNA-free p53 are presented at different temperatures in figure 4 for the WT and Q248 mutant. The flexibility profiles are strikingly similar, indicating that the overall structure of the DBD backbone is unchanged upon binding. Highly flexible regions of the unbound DBD are located in loops L1 and L2 and the turn connecting S7 and S8. Consistent with this observation, residues of the S7–S8 region, and Val-225 in particular, have been highlighted as inherently flexible parts of the human WT p53 DBD during a crystallographic analysis of the DNA-free conformation.[25]

Differences between the holo- and apo- structures reside in the flexibility of the loop L1 (residues 116–120) and the loop preceding helix H2 (residues 273–277). The rigidity of the loops is enhanced when p53 is bound to DNA. These observations are in excellent agreement with reported X-ray structural data.[34] Comparison of a DNA-free crystal structure of mouse p53 with that of DNA-bound DBD of human p53 has revealed similar minor structural differences between the two conformations, mainly around loop L1. Two contacts were identified between p53 backbone atoms and DNA at Lys-120 and Ala-276.[34] Both residues participate in direct interactions with DNA during the simulations, as confirmed by hydrogen bond and binding free energy analyses. In addition, conformational changes upon binding of WT p53 to DNA have been inferred from antibody binding measurements using PAb1620. In agreement with the observed changes in the flexibility profile, the epitope of PAb1620 has been isolated to residues 106–114 and 146–156,[35] although residues 145–157 and 201–212 have been characterized as the epitope in a conflicting report.[36] Because the computed main-chain flexibility profiles reflect changes in the slow collective dynamics, the existing experimental data and the present findings suggest that interactions between p53 and DNA at Lys-120 and Ala-276 also lead to the collective rearrangement of atoms in the DBD. In a separate comparative analysis of human WT p53 DBD in DNA-bound and unbound forms, Wang *et al.* detected differences in the flexibility of the S7-S8 turn, in addition to loops L1 and L2.[34] Changes are not observed in the turn region during MD simulation on neither short nor longer time scales. Discrepancies between simulated and crystallographic data may be due to crystal packing or limitations of force fields.

**Figure 5:**
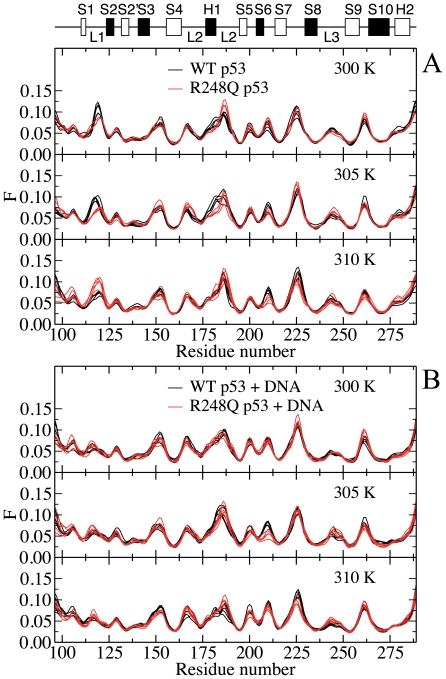
Effect of mutation on the main-chain flexibility profiles of (A) DNA-free(B) DNA-bound p53 at various temperatures. Flexibility profiles determined from Cα atoms of DNA-free and DNA-bound p53 comparing WT (black solid line) and R248Q (red solid line) variants at 300, 305 and 310 K.

Interestingly, qualitative features of the flexibility profiles are retained at all temperatures for both the WT and R248Q mutant p53, as illustrated in figure 5. More importantly, the main-chain flexibility of the L3 loop, which contains the mutated residue appears to be unaffected by the residue alteration. In the unbound form, differences between the main-chain flexibility of the WT and mutant p53 are localized to the highly flexible L1 and L2 loops, in agreement with the retention of the global fold in the mutant protein, as indicated by NMR spectroscopy.[20] The experiment, however, also revealed chemical shift changes between the native and mutant proteins in L2 and L3 as well as terminal residues of S4, S9 and S10. Analysis of the slow collective dynamics of Cα atoms showed no discernible differences in the β-strands and loop L3.

Upon binding to DNA, the loops L1 and L2 are stabilized and the structural plasticity becomes comparable in these regions. The stabilizing effect of DNA against misfolding and aggregation of p53 DBD has been reported previously.[15] The lack of sensitivity of the main-chain flexibility profiles of the p53-DNA complexes to the R248Q mutation confirms that the global structure of the DBD is essentially unaltered, as expected for a contact mutant. Changes in temperature seem to have little effect on the relative flexibility of the main-chain, suggesting that the global dynamics of the DBD may be insensitive to differences in temperature over the range investigated. These observations are consistent with the retention of major groove contacts in loops L1 and L3 upon mutation and changes in temperature, as revealed by the hydrogen-bonded networks of dominant p53-DNA clusters p53-DNA, and binding energy calculations.

**Figure 6:**
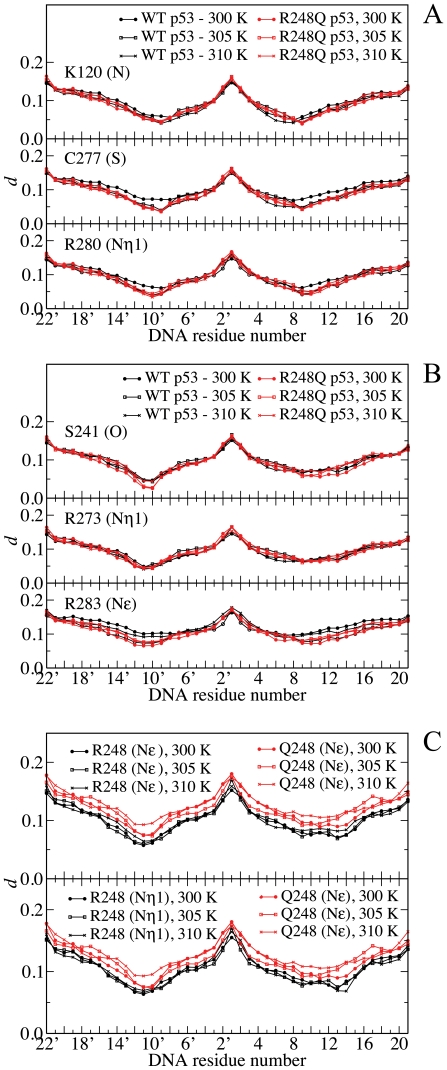
Plot of the dynamic correlation between DNA and contact sites. Correlation between DNA and selected side-chain atoms of p53 involved in (A) major groove, (B) phosphate and (C) minor groove contacts for WT (black solid line) and R248Q p53 (red solid line). Small values of *d* represent a strong correlation between atoms, while large values indicate weak correlation. For arginine residues, Nη1, Nη2, and Nε atoms of the guanidinium group give rise to similar plots – a single atom is depicted for each residue, except for the minor groove contact. Each data point corresponds to an average over 5 equilibrated trajectory segments of 0.2 ns.

So far, the slow collective motions of p53 molecules and their DNA-bound complexes have been analyzed in terms of their main-chain dynamics. To determine the role of side chains in the long-lived interactions between p53 and DNA, the correlation descriptor *d* was computed between interfacial side-chain atoms of p53 molecules and DNA residues (see Materials and Methods). By definition, the correlation descriptor is determined as a measure of binding between macromolecular partners. In particular, small values of d are associated with coherent dynamics between p53 and the DNA strand and are representative of strong intermolecular interactions in general. On the other hand, larger *d* values represent relatively independent motion and may be interpreted as weaker binding. Plots of the correlation descriptor between selected interfacial atoms of the side chains and each DNA residue are shown in figure 6. The plots share similar features - the largest levels of *d* occur at the DNA terminal residues, which are unbound to p53 DBD. Two minima, related to contact points on DNA, have also been identified. The minimum values of *d* are roughly comparable when p53 residues bind both strands of the DNA duplex, as observed for side-chains of Lys-120 in figure 6-A or Arg-248 in figure 6-C. For residues that display a net preference for contact at a particular strand, the plots tend to be asymmetrical with the lowest point corresponding to the preferred contact site.

Figures 6 illustrates the levels of correlation between p53 side chains and DNA, grouped according to the type of interactions, *i.e.*, major groove, phosphate and minor groove contacts, respectively [34]. As shown in Figures 6-A and 6-B, the correlation between DNA and p53 side-chains atoms involved in major groove and phosphate contacts of WT and R248Q p53-complexes are comparable at all temperatures.

This observation indicates that temperature or R248Q mutation has little influence on the correlated motion of these side chains, in accordance with our findings from the hydrogen-bonding and main-chain flexibility analysis. Arg-283 represents an anomaly within this trend: mutant p53-DNA complexes are characterized by low levels of correlation between atoms of DNA and guanidinium of Arg-283 relative to the WT complex at 300 and 310 K. Residue 283 is located near the C-terminus of the DBD and was not identified as a major participant in DNA binding based on the results of structural and thermodynamic calculations. Previous application of the essential collective dynamics approach has shown that terminal residues can give rise to artifacts in the subspace spanned by essential coordinates due to their artificially high mobility during MD simulations[37] ^,^ [38]. The behavior displayed by the side chain of Arg-283 may reflect a similar phenomenon. Plots of the correlation descriptor involving DNA atoms and the minor groove contact site R248/Q248 are markedly different from those of other contact side chains. figure 6-C indicates that correlations between DNA atoms and the amide group of Gln-248 are stronger than the corresponding correlations to the guanidinium group of Arg-248 (Nε and Nη atoms), especially at 305 and 310 K. At 300 K, the difference between the DNA-p53 side-chain correlations of Gln-248 and Arg-248 is less pronounced, especially in the vicinity of the contact points. Because the side chain of Arg-248 is involved in critical contacts with the minor groove, the differences may be indicative of altered binding characteristics. These observations are consistent with the changes in the hydrogen bonding network and stability. As described above, hydrogen bond analysis on representative clusters has identified direct intermolecular interactions involving Q248 at the DNA-p53 interface at 300 K but not at higher temperatures. In addition, binding energy decomposition has indicated that Glu-248 is the largest contributor to the destabilization of the mutant p53-DNA complex.

The present analysis of the collective essential modes of motion reveals that the slow local dynamics of critical interactions at the contact mutation site are also modulated by temperature. However, the long-living motions of side chains at the major groove contact sites (Lys-120, Cys-277 and Arg-280) are less sensitive to temperature changes and may provide an explanation for the apparent insensitivity of the main-chain dynamics to temperature increases.

### Therapeutic implications of restoring the R248Q mutant

The current work has confirmed that the introduction of the R248Q mutation into the p53 DBD affects the local structure and binding affinity at residues located far from the mutation site. Analysis of the temperature-sensitive behaviour of the R248Q mutation revealed that DNA binding was governed by a few critical residues and provides molecular evidence of the loss of binding at physiological temperatures. The findings can be applied to design a rescue strategy for the mutant structure. Relying on biophysical and structural data, Bullock *et al.* proposed that p53 mutations of the DBD could be divided into five classes. According to their classification, the R248Q mutant belonged to class I mutants along with the other DNA contact mutant R273H.[8] The remaining classes involved residues located in the DNA-binding portion as well as the ß-sandwich and the zinc-region of the DBD. Within class I, the two mutants presented different characteristics in the folded state: while the R273H retained the native fold, the R248Q had a distorted conformation relative to the WT p53.[8] Consequently, further investigations may be required before the findings can be generalized to both class I mutants or to other classes. Nonetheless, the R248Q mutation is highly relevant to human cancers. As listed above, it is the most frequent p53 mutation in numerous cancer. A typical search of the IARC database [39] establishes the prevalence of R248Q as a somatic mutation in 847 different tumors and as a germline mutation in 19 families with Li Fraumeni syndromes. Furthermore, as indicated above, this mutation is the most frequent p53 mutation in breast, colon, head, neck and skin cancers. It also ranks as the second most p53 mutation in esophageal, gastric, lung, ovarian, and prostate cancers. Therefore, it can be concluded that a potent and specific drug that can restore the R248Q mutant into the wild-type structure offers a great promise of activating the p53 pathway and, hence, turning on the apoptosis machinery in these types of cancer.

## Materials and Methods

### Generation of the mutated structure

Initial atomic coordinates for the DNA-bound WT p53 were obtained from the crystal structure[6] of the DBD in complex with a 21 base-pair DNA duplex (5′-ATATTTGGGCAAGTCTAGGAA-3′), available from the Protein Data Bank (PDB ID: 1TSR; chain B). The starting structure of the R248Q p53-DNA complex was generated from the corresponding WT complex using the software DeepView (Swiss PDB Viewer).[40] The orientation of the glutamine side chain was chosen so as to minimize steric clashes and maximize hydrogen bond interactions with neighboring residues. The initial coordinates of the DBD of the WT and R248Q p53 were used for DNA-free simulations.

### Molecular Dynamics Simulations

The WT and mutant p53 structures both with (holo) and without (apo) DNA were subjected to different MD simulations at temperatures of 300, 305 and 310 K employing the software NAMD[41] at physiological pH (pH 7) using the all-hydrogen AMBER99SB force field.[42] Protonation states of all ionizable residues were calculated using the program PDB2PQR.[43] The three cysteine residues along with the histidine residue that are coordinated to the Zn^2+^ ion were deprotonated. The resulting structures and their co-crystallized water molecules were immersed in the center of a TIP3P water cube after adding hydrogen atoms to the protein, DNA and water structures. The cube dimensions were chosen to provide at least a 20Å buffer of water molecules around the system. To neutralize and prepare the protein or protein-DNA complexes with a physiological ionic concentration (150 mM), chloride and sodium ions were introduced by replacing water molecules having the highest electrostatic energies on their oxygen atoms. The fully solvated systems were then minimized and subsequently heated to the simulation temperatures with heavy restraints placed on all backbone atoms. Following heating, the systems were equilibrated using periodic boundary conditions for 100 ps and energy restraints were reduced to zero in successive steps of the MD simulations. The simulations were then continued for ∼10 ns and atomic coordinates were collected over the final ns at intervals of 0.1 ps for subsequent collective dynamics analysis and binding energy calculations.

### Clustering analysis protocol

**Figure 7:**
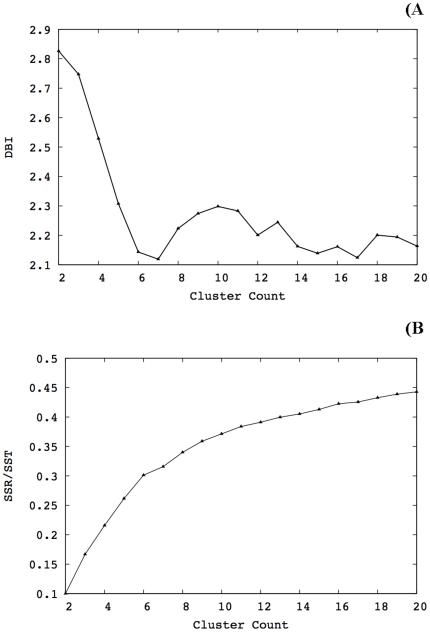
Clustering analysis for the wild type at 300 K. DBI exhibited local minima at cluster counts of 7, 12, 15 and 17. The percentage of variance explained by the data (sum of squares regression/total sum of squares, *i.e.*, SSR/SST) started to plateau after 14 clusters. Therefore, 17 clusters were extracted from the trajectory.

To generate a reduced set of representative p53-DNA models, we carried out RMSD conformational clustering with the average-linkage algorithm as implemented in the PTRAJ utility of AMBER10 using cluster counts ranging from 2 to 20 clusters. The application of the average-linkage algorithm, among other clustering algorithms, to MD trajectories has been validated in recent studies.[44] The clustering was performed on a set of structures extracted at 2 ps intervals over the last ns of the simulations. All C-alpha atoms were RMSD-fitted to the minimized initial structure in order to remove overall rotation and translation. RMSD-clustering was performed on the protein residues that constitute the DNA-binding interface. These residues were clustered into groups of similar conformations using the atom-positional RMSD of the entire amino acid, including side chains and hydrogen atoms, as the similarity criterion. The optimal numbers of clusters were chosen after evaluation of the Davies-Bouldin index (DBI)[45] and the “elbow criterion”[44] clustering metrics for different cluster counts. A high-quality clustering scheme is expected when high DBI values are calculated. On the other hand, using the elbow criterion, the percentage of variance explained by the data is expected to plateau for cluster counts exceeding the optimal number.[44] Using these metrics, a local minimum for DBI and a horizontal line for the percentage of variance explained by the data are expected for adequate clustering when the number of clusters is varied. figure 7 illustrates the outcome of clustering analysis using the DBI and elbow criterion at 300 K for the WT protein. Local minima can be identified in the DBI plot at cluster counts of 7, 12, 15 and 17. However, as the corresponding percentage of variance explained by the data started to plateau after 14 clusters, we concluded that 17 clusters is a reasonable cutoff to extract structures from the trajectory. The same procedure was followed in all simulations. Table 3 summarizes the results of clustering analysis for the rest of the simulations. The centroid of each cluster, the structure having the smallest RMSD to all members of the cluster, was chosen as the cluster representative structure and the dominant structures were used for hydrogen bonding analysis.

**Table 3:** Summary of the clustering analysis.

Structure	Temperature (K)	Optimal number of clusters	Population of largest cluster
Wild type	300	17	25%
	305	10	31%
	310	12	28%
Mutant	300	14	24%
	305	7	40%
	310	14	27%

### Hydrogen Bonding Analysis

Hydrogen bond analyses were performed using the visualization software VMD[46] on the dominant structures extracted from clustering analysis. A hydrogen bond was defined by a cutoff distance of 3.5Å between a donor and acceptor atom and an absolute angular deviation below 50° from linearity.

### Binding Energy Calculation and Decomposition

The binding energy between p53 and DNA was calculated using the molecular mechanics Poisson-Boltzmann surface area (MM–PBSA) method, introduced by Kollman *et al.* [47] In this work we used the method as implemented in the software AMBER 10.[34] That is, the total free energy is the sum of average molecular mechanical gas-phase energies (E_MM_), solvation free energies (G_solv_), and entropy contributions (-TS_solute_) of the binding reaction:
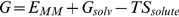
(1)


The molecular mechanical (*E*_MM_) energy of each snapshot was calculated using the SANDER module of AMBER10 with all pair-wise interactions included using a dielectric constant (ε) of 1. The solvation free energy (*G*_solv_) was estimated as the sum of electrostatic solvation free energy, calculated by the finite-difference solution of the Poisson–Boltzmann equation in the Adaptive Poisson-Boltzmann Solver (APBS) and non-polar solvation free energy, calculated from the solvent-accessible surface area (SASA) algorithm. The solute entropy was approximated using normal mode analysis. Applying the thermodynamic cycle for each protein-DNA complex, the binding free energy could be approximated by:
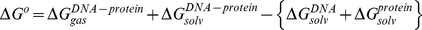
(2)


Here, (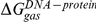) represents the free energy per mole for the non-covalent association of the DNA-mutant complex in vacuum (gas phase) at a representative temperature, while (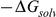) stands for the work required to transfer a molecule from its solution conformation to the same conformation in vacuum (assuming that the binding conformation of the DNA-protein complex is the same in solution and in vacuum). The total molecular mechanical energies could be further decomposed into contributions from electrostatic (*E*_ele_), van der Walls (*E*_vdw_) and internal energies (*E*_int_):
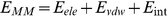
(3)


### Essential collective dynamics

The multi-scale dynamics of DNA-p53 complexes were analyzed according to the theory of essential collective dynamics.[34] First, essential coordinates were determined by applying principal component analysis (PCA) to each equilibrated trajectory. The covariance matrix was constructed from the mean square displacement of atomic positions over the course of 0.2 ns segments of the trajectories as follows:
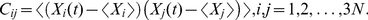
(4)


In the above equation, *X_i_* (*t*) denote the time-evolved Cartesian coordinates of atom *i* and *N* is the total number of atoms in the p53-DNA complex. Subsequent matrix digitalization yields eigenvalues λ*^k^* (*k* = 1, 2,…,3*N*) and their corresponding eigenvectors (or principal components) 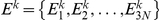. Together the normalized eigenvectors represent the intrinsic collective coordinates of the complex in configuration space and the eigenvalues correspond to the mean-square displacements along these coordinates. The collective degrees of freedom were ranked according to the magnitude of their corresponding eigenvalues and segregated into two orthogonal subspaces. A truncated set of collective degrees of freedom {*E* ^1^, *E* ^2^,…,*E* ^20^} associated with the 20 largest displacements {λ^1^, λ^2^,…,λ^20^} and representing at least 80% of the total displacement, was identified as the essential degrees of freedom. The complementary set of coordinates {*E* ^21^, *E* ^22^,…,*E* ^3*N*^ } can be attributed to small fluctuations in the motion of the p53-DNA complexes and was excluded from subsequent analyses. Unlike existing analyses of collective modes of molecular motion, in the present theory[19] the essential collective coordinates are used as dynamical variables in the Mori projection operator formalism[37],[38] to derive generalized Langevin equations. The resulting description of the collective conformational dynamics provides a means of identifying dominant dynamical correlations from short trajectory segments (0.2 ns long).[19] By grouping together individual contributions to essential collective coordinates of each atom such that 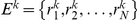, where 
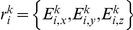
, it is possible to identify dynamical correlations from the atomic distribution in the subspace of essential motions. In particular, the distance between atoms in the 3*K*-dimensional space of essential motions represents the extent of dynamic correlation between them and does not depend on their locations in Cartesian coordinates. Dynamically correlated atoms are located within close proximity of one another in the subspace. Conversely, a relatively large separation between atoms is indicative of weakly correlated atomic motion. Therefore, the correlation descriptor, *d*, was defined as the distance between pairs of atoms *i* and *j* in the 3*K*-dimensional subspace of essential motions as follows:
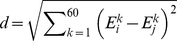
(5)


*d* was computed between selected interfacial side-chains atoms of p53 and all atoms of the DNA molecule in order to probe the local changes in the slow dynamics. For the sake of clarity, a single value of *d* (corresponding to the maximum correlation) is plotted for every DNA residue. The qualitative features of the plots are unaffected by this choice.

The slow backbone dynamics of the DBD of p53 molecules was investigated by computing their main-chain flexibility profiles. The flexibility, *F*, of individual Cα atoms was determined from the distances between their essential collective coordinate 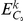
and the centroid ε*^k^* calculated over the coordinates of all Cα atoms, *i.e.*, [32]

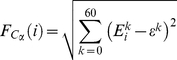
(6)


where, 
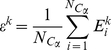
(7)


and 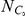
is the number of Cα atoms and *i* is the index running over them. The calculations were performed for both DNA-free and DNA-bound p53 molecules, in order to identify changes in the conformational dynamics upon DNA-binding.
